# Genome-wide analyses of long noncoding RNA expression profiles in lung adenocarcinoma

**DOI:** 10.1038/s41598-017-15712-y

**Published:** 2017-11-10

**Authors:** Zhenzi Peng, Jun Wang, Bin Shan, Fulai Yuan, Bin Li, Yeping Dong, Wei Peng, Wenwen Shi, Yuanda Cheng, Yang Gao, Chunfang Zhang, Chaojun Duan

**Affiliations:** 1 Institute of Medical Sciences, Xiangya Hospital, Central South University, Changsha, 410008 P. R. China; 2 Washington State University, Elison S Floyd College of Medicine, P.O. Box 1495, Spokane, WA 99210-1495 USA; 3 Department of Thoracic Surgery, Xiangya Hospital, Central South University, Changsha, 410008 P. R. China

## Abstract

LncRNAs have emerged as a novel class of critical regulators of cancer. We aimed to construct a landscape of lncRNAs and their potential target genes in lung adenocarcinoma. Genome-wide expression of lncRNAs and mRNAs was determined using microarray. qRT-PCR was performed to validate the expression of the selected lncRNAs in a cohort of 42 tumor tissues and adjacent normal tissues. R and Bioconductor were used for data analysis. A total of 3045 lncRNAs were differentially expressed between the paired tumor and normal tissues (1048 up and 1997 down). Meanwhile, our data showed that the expression NONHSAT077036 was associated with N classification and clinical stage. Further, we analyzed the potential co-regulatory relationship between the lncRNAs and their potential target genes using the ‘cis’ and ‘trans’ models. In the 25 related transcription factors (TFs), our analysis of The Cancer Genome Atlas database (TCGA) found that patients with lower expression of POU2F2 and higher expression of TRIM28 had a shorter overall survival time. The POU2F2 and TRIM28 co-expressed lncRNA landscape characterized here may shed light into normal biology and lung adenocarcinoma pathogenesis, and be valuable for discovery of biomarkers.

## Introduction

Lung cancer is the leading cause of death among all types of cancer^1^. Lung cancer has two histological types: small cell lung cancer (SCLC) and non-small lung cancer (NSCLC). The most common histological type of NSCLC is lung adenocarcinoma (ADC) that accounts for approximately 50% of NSCLC^2^. In general, ADC, originated from distal airways, is less frequently associated with chronic inflammation and smoking than squamous cell carcinoma (SCC)^3^. There is still no definitive biomarker for diagnosis and prognosis of ADC although several studies have identified a large number of genes associated with tumor initiation and progression.

LncRNAs have emerged as important regulators of physiology and disease. However, little is known about the mechanism of their functions^4^. lncRNAs are noncoding RNAs that are longer than 200 nucleotides in length. Recent RNA-SEQ based transcriptome studies revealed that 68% transcripts of human are lncRNAs and approximately 80% of them are unannotated^5^. lncRNAs are categorized into four groups: intronic, exonic, overlapping, and intergenic according to their locations in the genome^6^. lncRNAs are also classified as ‘cis’ or ‘trans’ lncRNAs according to their modes of regulation of transcription. lncRNAs can act as decoys, scaffolds, sponges, and guide of the protein and RNA molecules in cells^7^.

To delineate genome-wide ADC-associated lncRNAs expression, we employed locus-by-locus lncRNA and mRNA microarray probes to identify the lncRNAs and mRNAs that are differentially expressed between the ADC tumor tissues and the matched adjacent normal lung tissues. Our results establish a link between lncRNAs and clinical features of ADC, namely age, gender, smoking index, differentiation, TNM stage, and clinical stage. Using the ‘cis’ and ‘trans’ mode, we reveal potential co-regulatory relationship between the lncRNAs and their target genes. We also construct a “TFs-lncRNAs” two-element network and a “TFs-lncRNAs-mRNA” three-element network in ADC using hyper geometric distribution. We also identify potential cis-regulatory target genes of the differentially expressed lncRNAs in ADC by screening the co-expressed genes located near the differentially expressed lncRNAs. We identify the TFs that potentially regulate the differentially expressed lncRNAs by combining analysis of the TCGA database and hyper geometric distribution.

## Results

### LncRNAs and mRNAs expression profile in ADC

**Figure 1:**
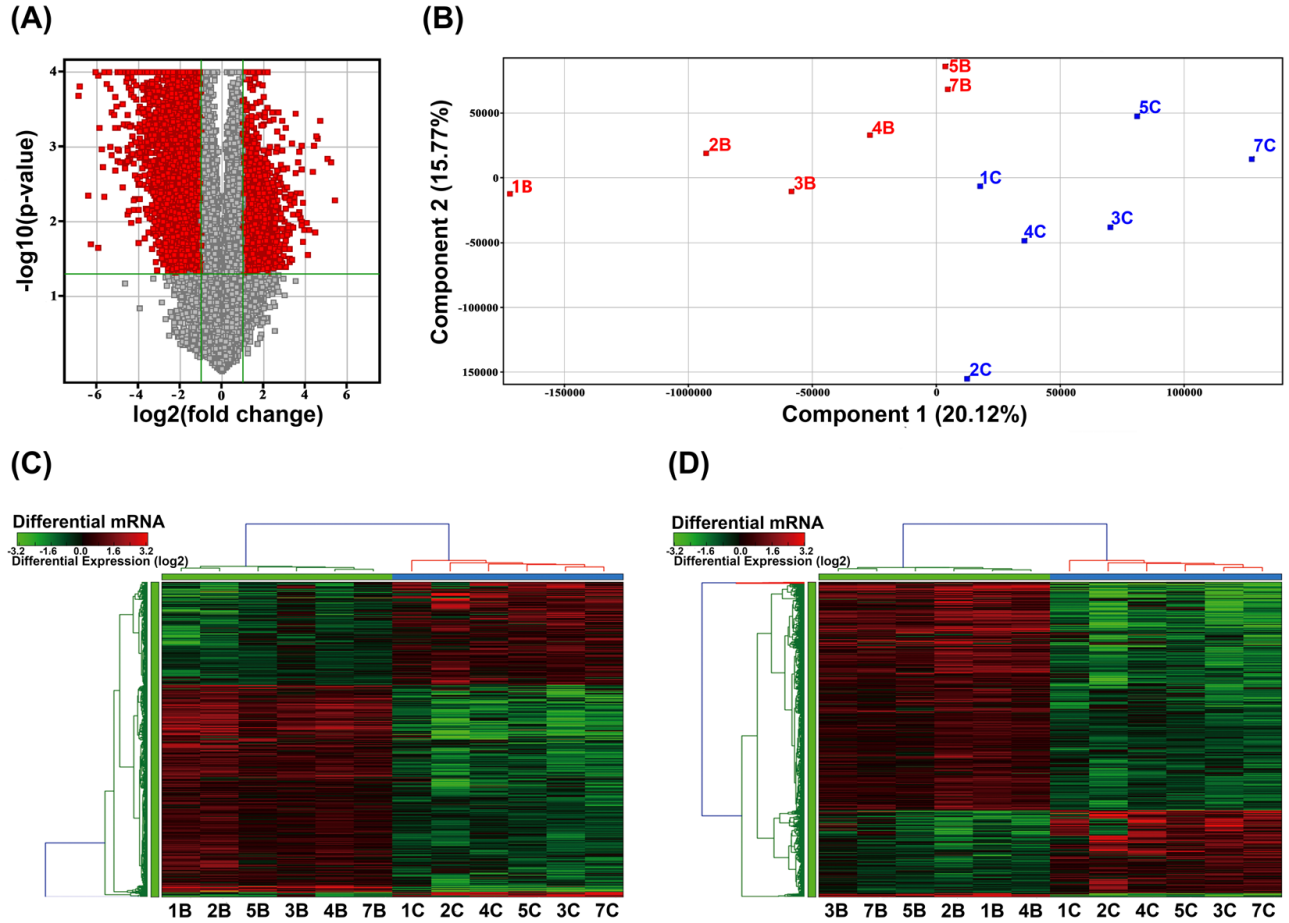
LncRNAs and mRNAs expression profiles in ADC. (**A**) Volcano Plot of the differentially expressed lncRNAs in ADC tumor tissues and adjacent normal lung tissues. The red points in the plot represent differentially expressed lncRNAs with statistical significance. (**B**) Principal Components Analysis. The B group (red plots) represents adjacent normal lung tissues and C group (blue plots) represents the ADC tumor tissues. (**C**) Hierarchical Clustering shows a distinguishable lncRNA expression profile and (**D**) mRNA expression profile.

To construct the lncRNA landscape in lung adenocarcinoma, we analyzed lncRNA and mRNA expression profile of 6 lung adenocarcinoma and their matched adjacent normal lung tissues using microarray. In order to avoid complicating factors derived from the heterogeneous nature of ADC clinical characteristics, we chose all female patients who have no smoking history and are between 45 and 55 year old. A volcano plot was used to provide an overview of the dysregulated lncRNAs in our microarray data sets (Fig. 1A). Principal component analysis (PCA) (Fig. 1B) and hierarchical clustering analysis (HCA) were applied to establish and cluster the lncRNA expression profile (Fig. 1C) and the mRNA expression profile (Fig. 1D). This difference distinguished the ADC group from the adjacent normal tissue group. From the microarray data, we identified 3045 lncRNAs and 2602 mRNAs that were differentially expressed between 6 paired ADC tumor tissues and adjacent normal lung tissues (Fold Change ≥2.0 or ≤0.5, p  < 0.05 and False discovery rate (FDR) <0.05=)^8^. Among these, 1048 lncRNAs and 694 mRNAs were upregulated in all six ADC samples, and 1997 lncRNAs and 1908 mRNAs were downregulated (see Supplementary Table S1, S2). Among them 57 upregulated lncRNAs and 329 downregulated lncRNAs were altered greater than 6.0-fold. The most upregulated lncRNAs was NONHSAT097328 (Fold change: 26.42) and the most downregulated was NR_038190.1 (Fold change: 117.37).

### Validation of differential lncRNA expression

**Figure 2:**
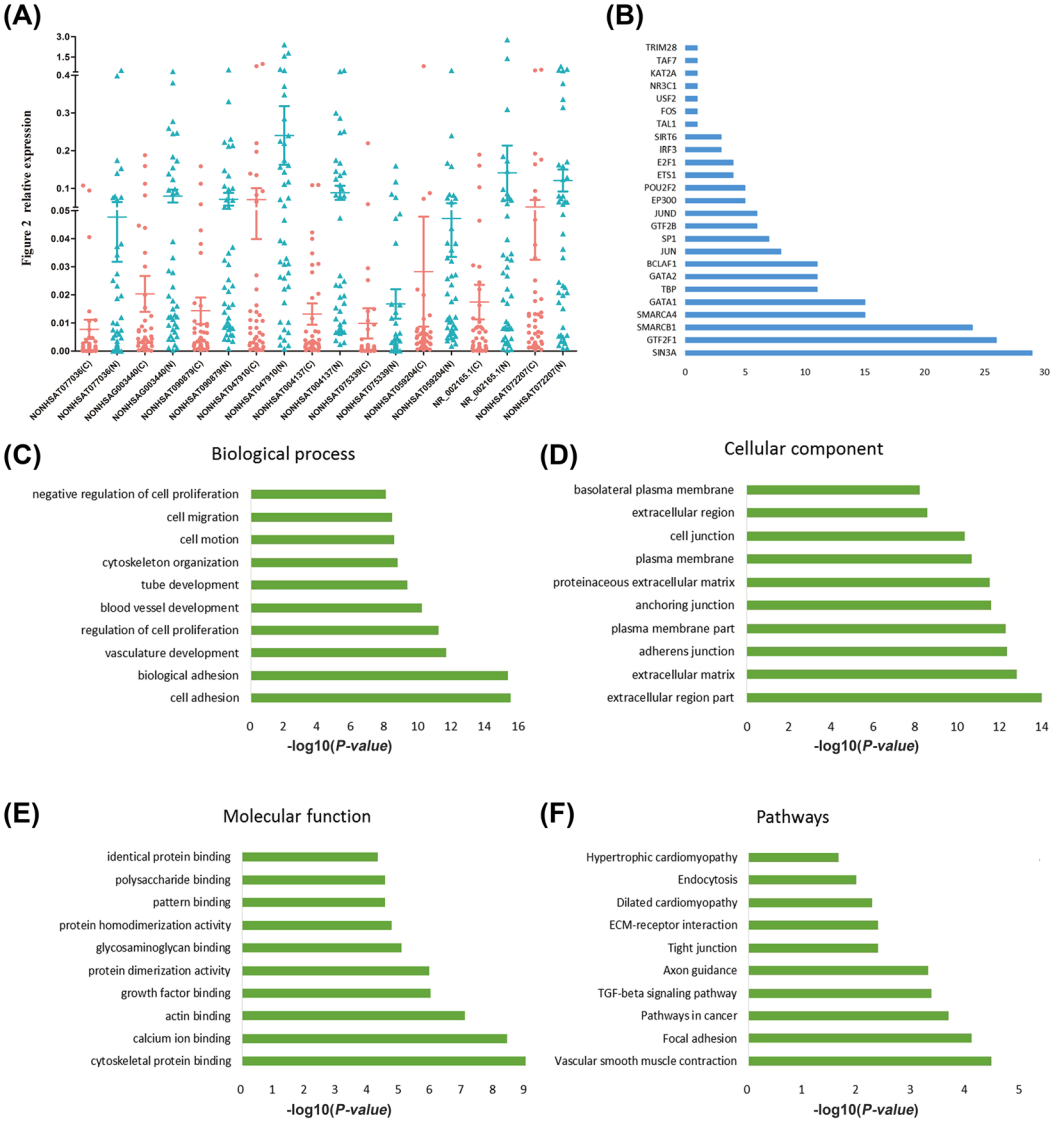
Validation of differential lncRNA expression and function. (**A**) Expression level of the selected lncRNAs in ADC tissues and adjacent normal tissues. qRT-PCR were used to verify differentially expressed lncRNAs (P ≤ 0.05). Gene expression was normalized to 36B4 expression. The blue points represent cancer tissues and the red points represent adjacent normal tissues. (**B**) Frequency distribution of lncRNAs enrichment on TFs. The X-axis is frequency distribution and Y-axis is the TFs name. (**C**–**F**) Go and Pathway analysis of lncRNAs co-expressed genes. The top 10 enriched terms were calculated as −log10 (P-value).

To evaluate our microarray performance, we measured the expression of 9 lncRNAs in all 42 paired ADC tumor tissues and adjacent normal lung tissues using qRT-PCR (Fig. 2A). The result showed that the expression patterns of these lncRNAs were consistent with microarray data in ADC tumor tissues and adjacent normal tissues.

To further investigate an association between lncRNAs expression and clinicopathological features in ADC, the patients were categorized by age, TNM stages, and differentiation. Based on the expression in paired tumor and adjacent normal tissues, we found NONHSAT077036 expression was associated with N classification and clinical stage (Table 1 P = 0.002, P = 0.008). Then we further investigated whether the association between NONHSAT077036 and TNM staging was specific to ADC. We measured the expression of NONHSAT077036 in 30 paired SCC tumor tissues and adjacent normal lung tissues using qRT-PCR. The data showed that NONHSAT077036 was not associated with N classification and clinical stage in SCC (Table 2 P = 0.253, P = 0.448). This indicated that NONHSAT077036 was only associated with ADC but not with SCC.

**Table 1:** *P*-value of lncRNAs expression with clinicopathological features in ADC patients.

LncRNAs No.	Age (years)	Gender	Smoking Index	Differentiation	T classification	N classification	Clinical Stage
NONHSAT077036	0.809	0.55	0.056	0.242	0.101	0.002	0.008
NONHSAG003440	0.96	0.94	0.461	0.64	0.894	0.399	0.919
NONHSAT090879	0.497	0.591	0.139	0.872	0.847	0.259	0.815
NONHSAT047910	0.114	0.048	0.117	0.682	0.705	0.607	0.823
NONHSAT004137	0.96	0.94	0.461	0.379	0.848	0.399	0.919
NONHSAT075339	0.188	0.55	0.244	0.946	0.646	0.853	0.906
NONHSAT059204	0.96	0.289	0.233	0.39	0.482	0.701	0.21
NR_002165.1	0.748	0.127	0.755	0.966	0.763	0.701	0.925
NONHSAT072207	0.957	0.824	0.102	0.436	0.285	0.091	0.269

**Table 2:** *P*-value of lncRNAs expression with clinicopathological features in SCC patients.

LncRNAs No.	Age (years)	Gender	Smoking Index	Differentiation	T classification	N classification	Clinical Stage
NONHSAT077036	0.569	0.612	0.688	0.139	0.574	0.253	0.448
NONHSAG003440	0.87	0.014	0.999	0.275	0.041	0.589	0.035
NONHSAT090879	0.424	0.026	0.119	0.378	0.999	0.084	0.378
NONHSAT047910	0.688	0.043	0.261	0.154	0.147	0.845	0.083
NONHSAT004137	0.146	0.645	0.039	0.495	0.466	0.157	0.347
NONHSAT075339	0.596	0.18	0.457	0.422	0.142	0.548	0.105
NONHSAT059204	0.034	0.502	0.352	0.422	0.142	0.548	0.704
NR_002165.1	0.289	0.18	0.457	0.422	0.834	0.489	0.704
NONHSAT072207	0.71	0.814	0.473	0.99	0.085	0.969	0.683

**Figure 3:**
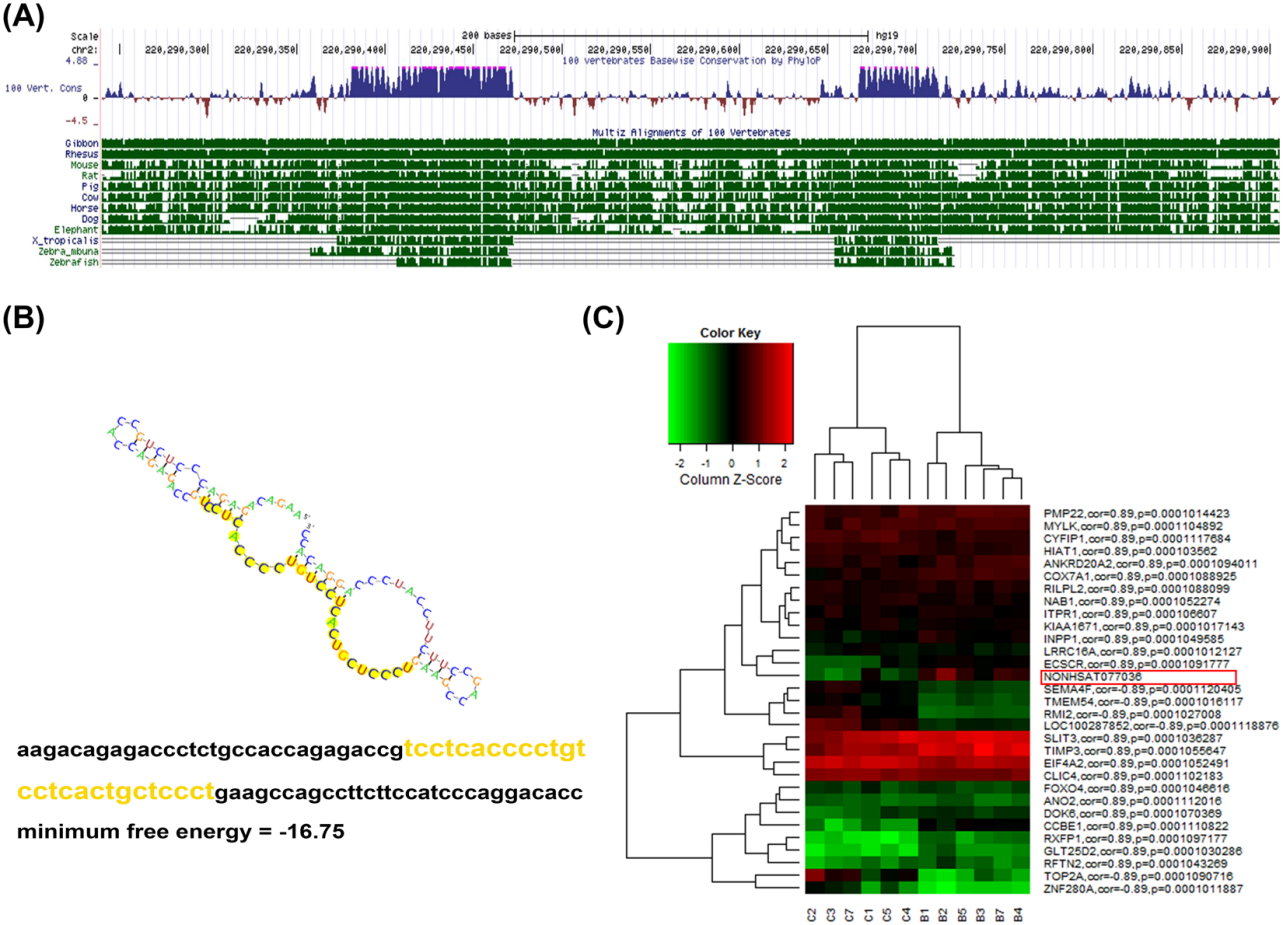
Function prediction of lncRNAs NONHSAT077036. (**A**) Sequence conservation of NONHSAT077036 from humans to zebrafish. (**B**) Sequence similarity between NONHSAT077036 and H19. (**C**) Potential target genes of NONHSAT077036.

To gain further insight into the role of NONHSAT077036 in ADC, we examined its sequence and structure across species. Generally, lncRNAs are not as conserved as protein coding genes. Therefore, it is difficult to predict lncRNA function based on evolutionary conservation (Fig. 3A). However, NONHSAT077036 showed strong sequence conservation from zebrafish to human, which suggests important functions of NONHSAT077036. Intriguingly NONHSAT077036 exhibited sequence similarity to H19 (132–159, 28 bp), which is also elevated in lung cancer and promotes cancer cell proliferation^9^ (Fig. 3B). We then predicted the target genes of NONHSAT077036 using co-expression analysis (Fig. 3C). Among these predicted target genes, many are critical regulators of tumorigenesis. For instance, there is a significant negative correlation between NONHSAT077036 and TOP2A (r = −0.89) that is an oncogene involved in G2 checkpoint in response to DNA damage^10^.There is a positive correlation between NONHSAT077036 and CCBE1 (r = 0.89) that reported to regulate extracellular matrix remodeling and migration^11^. These correlations suggest regulation of proliferation and invasion by NONHSAT077036 in ADC.

### Identification of potentially functional lncRNAs in ADC

To predict potential functions of the differentially expressed lncRNAs, we calculated the correlation value of lncRNAs and mRNAs. Differentially expressed lncRNAs were divided into two subsets: the upregulated and downregulated lncRNAs. Top 200 most differentially expressed lncRNAs in each subset were selected for further analysis. LncRNA NR_038190.1 was the most significantly downregulated lncRNAs. The analysis pertaining to it was shown as a representative result. The top 30 correlations between lncRNA NR_038190.1 and its target genes were showed in Table 3.

**Table 3:** Target genes of NR_038190.1.

Gene Symbol	*p*-value	Correlation	Gene Symbol	*p*-value	Correlation
TBX4	0.000101	0.891002	PIK3C3	0.000107	0.889654
RAPGEF4	0.000101	0.891000	PTPLAD2	0.000107	0.889621
TTC28	0.000102	0.890780	SYNGR1	0.000107	0.889601
SH2D1B	0.000102	0.890723	ZAK	0.000108	0.889386
ITGA8	0.000102	0.890706	APPBP2	0.000109	0.889201
PIP5K1B	0.000102	0.890704	SPATA13	0.000110	0.888938
C1orf145	0.000102	0.890619	CBFA2T3	0.000110	0.888911
ANXA3	0.000103	0.890460	SNX1	0.000110	0.888899
GIMAP1	0.000103	0.890407	CCNDBP1	0.000111	0.888839
FAM83A	0.000103	−0.890375	NHSL1	0.000112	0.888607
BDNF	0.000104	0.890349	FIGF	0.000112	0.888513
PLEKHH2	0.000104	0.890150	WNT7A	0.000113	0.888409
FAM162B	0.000105	0.889993	KIDINS220	0.000113	0.888351
PRKG2	0.000105	0.889954	JPH4	0.000113	0.888299
CTDSP1	0.000106	0.889779	F10	0.000115	0.887960

We further analyzed the enrichment of GO (http://geneontology.org/) and KEGG pathway (http://www.kegg.jp/kegg/) terms associated with the lncRNAs that were differentially expressed between ADC and normal tissues. DAVID functional annotation software (https://david.ncifcrf.gov/home.jsp)^12^ was used to analyze all co-expressed genes. Top 200 upregulated lncRNA genes and 200 downregulated lncRNA genes were subjected to GO and KEGG pathway analyses. We selected the top 200 reliability prediction terms (according to the *p*-value and enrichment) for co-expressed and aberrant lncRNA genes, respectively. The top 200 terms in the GO terms were highly enriched for cell adhesion, proliferation, migration (ontology: molecular function), extracellular region part, adheren junction (ontology: cellular component) and cytoskeletal protein binding, growth factor binding (ontology: molecular function). Top 200 terms in the KEGG pathway were associated with pathways in cancer. The most significant top 10 GO terms and KEGG pathway are shown in Fig. 2C–F. Besides, we calculated the enrichment of functional terms of co-expressed genes for each differentiated lncRNAs (see Supplementary Table S3, S4).

### LncRNAs target prediction

**Figure 4:**
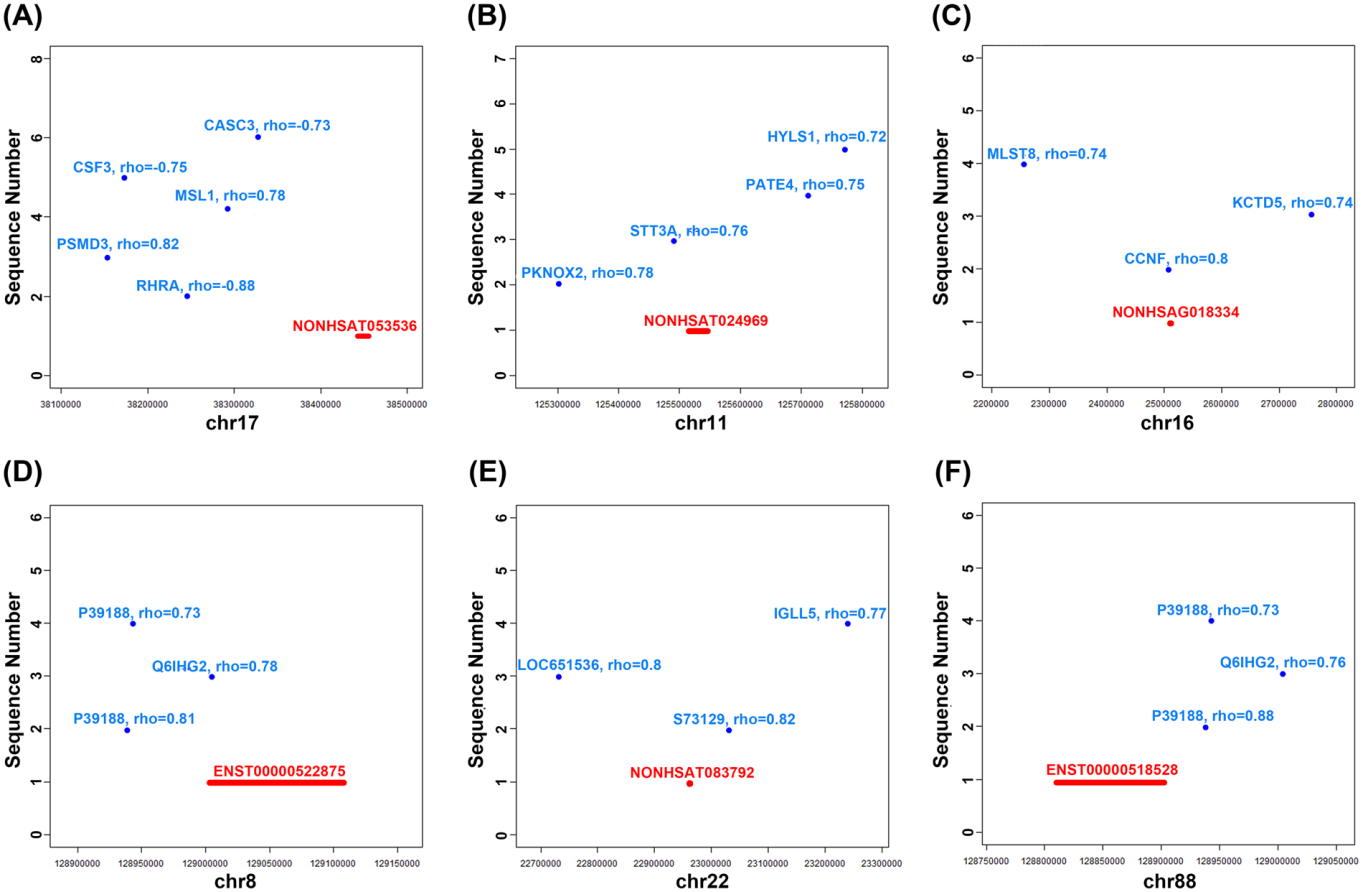
Cis-regulation genes of representative lncRNAs in the chromosome. The X-axis represents lncRNA position in chromosome, the Y-axis represents correlation coefficient of lncRNA and potential “cis” genes. The red line represents the genome width of lncRNA and blue point represents the position of potential “cis” genes.

To explore how lncRNAs function in lung adenocarcinoma, we predicted the cis- and trans-regulated genes of the differentially expressed lncRNAs using co-expression network analysis. The co-expressed genes within 300 kb upstream or downstream from a selected lncRNA were identified as potential “cis” genes of a given lncRNA (*p*-value of correlation <=0.05). We predicted the cis-regulated genes at the top of differentially expressed lncRNAs (see Supplementary Table S5). In ADC tissue and adjacent normal lung tissue controls, 56 upregulated lncRNAs had 92 ‘cis’ genes and 35 downregulated lncRNAs had 42 ‘cis’ genes. 8 upregulated lncRNAs had at least 3 ‘cis’ genes. The maximal number of cis genes assigned to a differentially expressed lncRNA was 5. The cis relationship of 6 significantly dysregulated (up.58_NONHSAT053536, up.106_NONHSAT024969, up.128_NONHSAG018334, up.129_ENST00000522875, up.190_NONHSAT083792, up.195_ENST00000518528) are shown in Fig. 4A–F.

Among all these potential cis-regulated target genes, further analysis showed that the most highly related categories were the processes that affect cell growth, differentiation, and migration. The most correlated genes were related to cell cycle. Among the ‘cis’ genes, EGR1, EHF, PLCE1, PKNOX2, MLST8, FGF17, FOSB, PTPRQ, NKTR, and CAV2 are known to function in cell growth and differentiation. CDK7, CCNF, CDKN3, CDC20, TUBG1, FEN1, and GRK5 are critical regulators of cell cycle. PEA15 and CAV2 are regulators of apoptosis among ‘cis’ genes. JAM3, CD36, and RAB21 are believed to bear a critical role in adhesive processes and cell migration. These cis-regulated lncRNAs are potential regulators of ADC.

**Figure 5:**
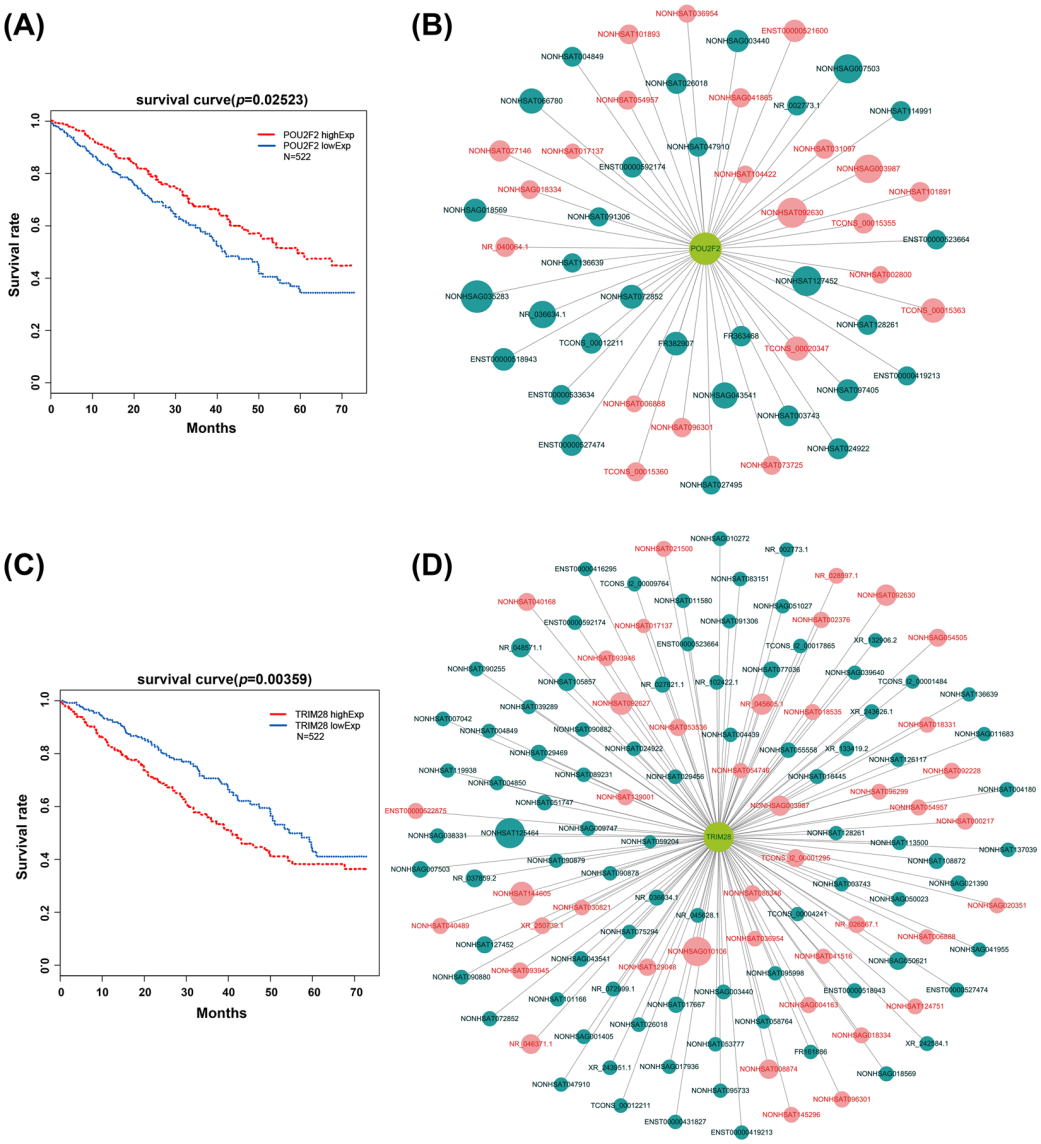
TFs and lncRNAs co-expressed network. (**A**,**C**) Kaplan-Meier survival analysis of POU2F2 and TRIM28 expression of ADC patients in TCGA database. (**B**,**D**) “POU2F2-lncRNAs” and “TRIM28-lncRNAs” two-element network. Green points represent up-regulated lncRNAs. Red points represent down-regulated lncRNAs. The size of each dot is proportional to the magnitude of the change of a given lncRNA.

It is noteworthy that lncRNAs also act in ‘trans’ to regulate TFs mediated chromatin remodeling and transcription^13^. We intended to discover which TFs might interact with the differentially expressed lncRNAs using hyper geometric distribution that can calculate the overlap of TFs target genes and chromatin regulators with the co-expressed lncRNA genes. As showed in Fig. 2B, the lncRNAs were significantly regulated by 25 TFs: SIN3A, POU2F2, TRIM28, etc. Early studies have demonstrated that these TFs are master regulators of cancer^14–20^. We further analyzed the relationship between the top 25 lncRNA related TFs and overall survival in TCGA database. We observed that patients with lower expression of POU2F2 and higher expression of TRIM28 had shorter overall survival time (Fig. 5A,C), suggesting that POU2F2 and TRIM28 are biomarkers for poor prognosis of ADC. Based on the results of the lncRNA co-expression analysis, we generated “POU2F2-lncRNAs” and “TRIM28-lncRNAs” two-element network by Cytoscape software (Fig. 5B,D). Then we added target co-expressed genes and generated “POU2F1-lncRNAs-mRNAs” and “TRIM28-lncRNAs-mRNAs” three-element relationship tables (see Supplementary Table S6).

## Discussion

Aberrant expression lncRNAs is recognized as a hallmark feature in pathogenesis and progression of various diseases, including lung cancer. ADC is the largest histological phenotypes of lung cancer. However, the genome-wide expression profile and classification and function of lncRNAs have not been examined in ADC. Unraveling the functions and mechanisms of these lncRNAs can substantially improve our understanding of ADC. Integration of lncRNAs and target genes profiling is also a promising approach to identify effective biomarkers of ADC. Therefore, we screened the genome-wide expression profile of lncRNAs and mRNAs in 6 paired ADC tumor tissues and adjacent normal lung tissues in this study. Microarray data were further validated by qRT-PCR in another 42 paired tissues. We predicted the function of selected lncRNAs according to co-expression genes and Gene Ontology (GO) biological process. In addition, we predicted ‘cis’ and ‘trans’ regulated modes of the 200 tops differentially expressed lncRNAs to find out how they might regulate ADC progression. Several dysregulated lncRNAs reported in the previous studies were also found in our study. The overlapping lncRNA expression profiles between our current study and the previous studies validate the significance of our study. For instance, lncRNA PVT1 is upregulated in various human cancers^21,22^, including lung cancer^23^. In our data, 13 probes were designed to measure PVT1 and all of them were upregulated. The average fold change was 5.45. More importantly, we showed that PVT1 may be regulated by 5 TFs: SIN3A, KAT2A, E2F4, E2F1 and GATA2. Another example is the tumor suppressor FENDRR. FENDRR inhibits cell proliferation and migration and is downregulated in cancer cell lines and cancerous tissues^24^. In support of this view, our results also showed a 22.57-fold decrease of FENDRR expression in ADC tissues. Recent reports indicated that FENDER overexpression suppressed invasion and migration by downregulating fibronectin1 expression. Our results also showed a significant inverse correlation between all 5 FENDRR probes and fibronectin1 (Correlation valued ≥0.7 or ≤−0.7). However, functions of the novel differentially expressed lncRNAs identified in our study remain to be characterized experimentally.

It is of note that lung cancer is a highly heterogeneous disease. Our analysis showed that NONHSAT077036 expression was associated clinicopathological features only in ADC patients (P < 0.05=, but not other histologic subtypes of non-small cell lung cancer. However, additional studies are needed to verify the significance of NONHSAT077036 in ADC. Functional analysis of NONHSAT077036 needs to be carried out in cell and animal based models of ADC.

We also performed Gene Ontology and pathway analysis of co-expressed genes of 400 lncRNAs. Our data indicate that the most related categories include cell adhesion, proliferation, migration, growth factor binding, etc. These GO terms are well-established critical factors in tumorigenesis and tumor progression.

Little is known about the exact function of lncRNAs, although evidence so far indicates that lncRNAs participate in various biological processes. We analyzed the aberrantly expressed lncRNAs in ADC in “cis” and “trans” regulated mechanisms. Our data indicated that cis-regulated target genes participate in initiation and progression of ADC. For instance, EGR1 belongs to the EGR family whose activation is involved in differentiation and mitogenesis. In addition, EGR1 supports FGF-dependent angiogenesis during neovascularization and tumor growth^25^. Another study indicates that EGR1 upregulates the expression of lincRNA H19 in liver cancer^26^. In addition, EHF encodes an ETS transcription factor that is expressed in epithelial-specific manner. It has been reported that EHF is silenced by epigenetic mechanisms during NSCLC (non-small cell lung cancer) development^27^. EHF in ovarian cancer cells regulates cell proliferation and G1 phase checkpoint^28^. It is noteworthy that our bioinformatical analysis of the lncRNAs was largely based on a correlation between the expression patterns of lncRNAs and mRNAs. Further studies are needed to experimentally validate the link between lncRNAs and mRNAs identified in the current study.

It is generally accepted that lncRNAs can directly interact with gene promoters and TFs. Many of these lncRNAs recruit protein factors to enhancers and regulate the activity of enhancers^29^. Transcriptional processes are also controlled by lncRNAs via their interaction with primary coding transcripts. A survey of correlation between lncRNAs and TFs also helps us reveal its function. TCGA database contains a large number of clinical information about ADC patients. We carried out survival analysis of 25 related ‘trans’ mode TFs in TCGA database and found that the patients with lower expression of POU2F2 have a shorter overall survival. These findings suggest that POU2F2 may be a biomarker for a poor prognosis of ADC. To date, the role of POU2F2 is controversial. Oct-2, encoded by the gene POU2F2, is a B-cell restricted transcription factor. Emerging evidence indicates that POU2F2 is essential to the later stages of B-cell differentiation^30^. Mice with deletion of POU2F2 die shortly after birth^31^. Recent evidence indicates that POU2F2 mediates metastasis induced by ROB01 in gastric cancer^32^. In our study, POU2F2 appears to be a tumor suppressor in ADC. Moreover the POU2F2-lncRNAs” two-element network modulates the expression of 53 lncRNAs. Deciphering the functions of POU2F2 in ADC needs further investigations. We identified another lncRNA related TF, TRIM28 in ADC in our study. The prevailing view is that knockdown of TRIM28 expression impairs cell proliferation in NSCLC cell lines. In addition, patients with elevated expression of TRIM28 suffered shorter tumor-specific survival^33^. Our data support this view because the patients with elevated expression of TRIM28 have a shorter overall survival. Moreover the TRIM28-lncRNAs” two-element network modulates the expression of 129 lncRNAs.

It is currently well accepted that molecular networks of multiple genes and pathways, instead of a single gene or a pathway, underlie pathogenesis of cancer. Network analysis has provided an efficient method to model biological processes. It should also be emphasized that dynamic feedback motifs will help us to obtain a unified view of various cellular processes^34,35^. Thus, it is necessary to integrate omics data (gene regulatory networks, cell signaling networks and metabolic networks) in network analysis. Network analysis is an effective approach in predicting potential lncRNA–disease associations. There are a wide range of computational models and web servers that have been developed for this purpose. Chen *et al*. introduced state-of-the-art computational and FMLNCSIM models to identify disease-related lncRNAs from experimental validation. They developed an effective computational models to construct lncRNA functional similarity and the similarity scores (Long non-coding RNAs and complex diseases: from experimental results to computational models) (FMLNCSIM: fuzzy measure-based lncRNA functional similarity calculation model)^36–40^.

An important limitation of our work is that we have not distinguished different sub-clones. Cancer is a highly heterogeneous disease although one clone can become the dominant population in the tumor at diagnosis^41^. There are many distinct sub-clones that coexist in a tumor and are likely derived from different clonal backup^42,43^. Thus, a drug targeting only one sub-clone within a tumor may have limited effect. Tumor genome-wide analysis is needed to model cancer cells by constructing networks for individual clones.

Our findings reported in the current study warrant further investigations of the mechanisms of the differentially expressed lncRNAs to understand their clinical significance in ADC. The lncRNA profile we established in ADC will lay the foundation for a better understanding of the impacts of lncRNAs in ADC patients. The ADC-associated lncRNAs identified in our study are promising biomarkers with potential in tumor diagnosis, classification, prognosis and therapeutic evaluation.

## Materials and Methods

### Samples

All lung adenocarcinoma patients were diagnosed at the Thoracic Surgery Department of Xiangya Hospital, Central South University. The cohort of 42 lung adenocarcinoma patients provided written informed consent in compliance with the code of ethics of the World Medical Association. The collection and usage of the clinical specimens were approved by the Xiangya Hospital Medical Research Ethics Committee. All experimental methods were performed in accordance with the relevant guidelines and regulations of Xiangya Hospital Medical Research Ethics Committee and the Scientific Research Project 201403216 (Histopathological Application). The tumor-node-metastasis was classified based on the criteria of the eighth tumor-node-metastasis (TNM) staging system. All tissues were stored at −80 °C after initial freezing in liquid nitrogen. The collection of clinical data of these patients include sex, age, smoking status, differentiation and TNM stage (see Supplementary S7).

### RNA extraction and array data production

Sample preparation and microarray hybridization were performed by OE Biotech Corporation, Shanghai, P.R. China. Briefly, total RNA was extracted and 200ng RNA was purified from each sample (RNasey Mini Kit (Qiagen p/n 74104). The Agilent 2100 bioanalyzer and RNA LabChip® kits was used to assess RNA quality. RNA Integrity Number (RIN) ≥7 and 28 S/18 S ≥0.7 was used for synthesizing double-stranded cDNA (see Supplementary Table S8). Then cDNA was labeled by Cy3-dCTP and hybridized to the OE Biotech Human 4 × 180 K lncRNAs chip, which contained 46,506 lncRNAs probes and 30,656 mRNAs probes collected from eight databases, including Agilent ncRNA, GencodeV13, lncRNAdb, H-invDB, RefSeq, NONCODE v3.0, UCR and UCSC lncRNAs Transcripts.

### qRT-PCR validation

qRT-PCR was used to validate our microarray data. Briefly, total RNA was extracted using Trizol reagent (Invitrogen) and then reverse-transcribed using GoScript™ Reverse Transcription System (Promega) in accordance with the manufacturer’s protocol. Real-time PCR was performed using All-in-One™ qPCR Mix (GeneCopoeia). Specific primers are listed in Table 4. Each sample was normalized by the internal control gene of 36B4. The results represent means of 3 repetitions and were quantified by the 2^−ΔΔct^ method. The mRNA levels of lncRNAs between tumor and non-tumor tissues were compared using T-test (P < 0.05) using R. The mRNA levels of lncRNAs between cancer and non-cancer tissues were compared using T-test (P < 0.05) using R. Pearson × 2 test was used to analyze the relationship between lncRNAs expression and clinicopathologic parameters in SPSS software (version 20.0, Chicago, IL).

**Table 4:** Primers Used for qRT-PCR Analysis of lncRNAs.

LncRNAs No.	Position	Primer sequence
NONHSAT077036	Forward	TGAAGAAGTAACAAGCCTGTCT
	Reverse	TGGTCTTGATCATCACCGTCT
NONHSAG003440	Forward	GGAGGAGTGTGGAGGTTCAA
	Reverse	TACATGCCTGGGTCAGCTAC
NONHSAT090879	Forward	ATATACTACAGTGCGTTGTTGTCC
	Reverse	AGCAGTTGGATGACAGAGAATAG
NONHSAT047910	Forward	CAAGTCCCAGAATCCTCCAG
	Reverse	AGGCTTACAGGAAATGTGCAG
NONHSAT004137	Forward	AGCCAGTCTAGTGGACAGAGA
	Reverse	CCTGCATTGAATAATCACAAGACCA
NONHSAT075339	Forward	GACTGGGTTTATTACCCTCTCCT
	Reverse	TAAGACTGCCTCTGCCCTTC
NONHSAT059204	Forward	GAGTGTGACCTAGCGCAGAA
	Reverse	GAGCACACCTTCCAAGCAC
NR_002165.1	Forward	ATGGCTAGAAGTGACCCCAG
	Reverse	TGCCCAGCCTAGACTTCTC
FR407620	Forward	CACCTCCCTCAAACCTGTCT
	Reverse	GCCAGAATTGCTTGCCTCAT
NONHSAT072207	Forward	TTGGGAGTGTGCATGAGGTA
	Reverse	TTTGGTTACATGTCGGCAGT

### Bioinformatics analysis

Data analysis including heat map, volcano plot, PCA and survival was carried out using R by gplots, lattice, MASS, ggplot2, hash and survival packages (https://www.R-project.org/)^44^.

### Differential expression analysis

Differentially expressed lncRNAs and mRNAs were identified using paired t-test (Fold Change ≥2.0 or ≤0.5, p < 0.05 and FDR < 0.05). The microarray data have been uploaded in NCBI Gene Expression Omnibus (GEO) and the GEO accession number is GSE85716. Red indicates high expression and green indicates low expression in tumor tissues.

### lncRNA co-expression analysis

We evaluated potential co-expression between lncRNAs and mRNAs using Pearson Correlation. A positive correlation between a lncRNA and a mRNA was defined as a Pearson Correlation greater than 0.7 and a *p*-value less than 0.05. Hypergeometric cumulative distribution function was used to calculate the enrichment of co-expressed mRNAs. The False Discovery rate was determined using the method as previously described. The ontology of co-expressed genes was categorized by gene annotation and summary information obtained from DAVID database^12^. Annotations of the lncRNAs co-expressed mRNAs were determined using GO analysis on cellular component, molecular function, biological processes and specific pathways.

### Prediction of lncRNAs function

We searched for an lncRNA co-expressed genes within a 300 kb window of each lncRNA in the top 200 up-regulated and downregulated lncRNAs (P < 0.05=. The co-expressed genes on both sides of an lncRNA were defined as potentially ‘cis’ regulated genes by a given lncRNA. To examine which genes were potentially ‘trans’ regulated by lncRNAs we determined which TF might interact with the lncRNAs of interest using Jemboss software. TF target gene sets were obtained from Encyclopedia of DNA Elements (ENCODE). Hyper geometric distribution was used to identify the overlap of TFs target genes and co-expressed genes of lncRNAs. *p*-value was used to measure the enrichment of differentially expressed genes in the term. The TF and lncRNAs relationship networks were drawn using Cytoscape software. The TFs survival analysis was performed using the RNA-Seq and survival data extracted from the TCGA database (https://portal.gdc.cancer.gov/).

## Electronic supplementary material

Supplymentary information

Dataset 1

Dataset 2

Dataset 3

Dataset 4

Dataset 5

Dataset 6

Dataset 7

Dataset 8
