# Supplementary material for: Genome-wide analyses of long noncoding RNA expression profiles in lung adenocarcinoma
Source: Sci Rep. 2017 Nov 10;7:15331. doi: 10.1038/s41598-017-15712-y (PMC5681506; doi:10.1038/s41598-017-15712-y)
Supplement: Supplementary file 1 — Supplymentary information [file 41598_2017_15712_MOESM1_ESM.pdf]

Genome-wide analyses of long noncoding RNA expression profiles in lung adenocarcinoma

Zhenzi Peng[1], Jun Wang[1], Bin Shan[2], Fulai Yuan[1], Bin Li[1], Yeping Dong[1], Wei Peng[1], Wenwen Shi[1], Yuanda Cheng[3], Yang Gao[3], Chunfang Zhang[3] and Chaojun Duan*[1,3]

[1]Institute of Medical Sciences, Xiangya Hospital, Central South University, Changsha 410008, PR China;
[2]College of Medical Sciences, Washington State University Spokane, WA 99201, USA.
[3]Department of Thoracic Surgery, Xiangya Hospital, Central South University, Changsha 410008, PR China;
*duancjxy@126.com

Supplementary information

Supplementary Table S1 Differentially expressed lncRNAs.
Supplementary Table S2 Differentially expressed mRNAs.
Supplementary Table S3 Each lncRNAs GO enrichment.
Supplementary Table S4 Each lncRNAs KEGG enrichment.
Supplementary Table S5 Cis-regulated genes of lncRNAs.
Supplementary Table S6 POU2F2_ and TRIM28_LncRNAs_Three-element relationship.
Supplementary Table S7 Clinical and phenotypical characteristics patients.
Supplementary Table S8 RNA quality assessment in the Agilent 2100 bioanalyzer.
